# Orbital Rosai-Dorfman disease initially diagnosed as IgG4-related disease: a case report

**DOI:** 10.1186/s40478-020-00995-6

**Published:** 2020-07-18

**Authors:** Nishanth S. Iyengar, Danielle Golub, Michelle W. McQuinn, Travis Hill, Karen Tang, Sharon L. Gardner, David H. Harter, Chandranath Sen, David A. Staffenberg, Kristen Thomas, Zachary Elkin, Irina Belinsky, Christopher William

**Affiliations:** 1 NYU Grossman School of Medicine, NYU Langone Health, 550 First Ave, New York, NY 10016 USA; 2 Department of Neurosurgery, Zucker School of Medicine at Hofstra/Northwell, Northwell Health, 300 Community Dr, Manhasset, NY 11030 USA; 3 Department of Neurosurgery, NYU Grossman School of Medicine, NYU Langone Health, 530 First Ave, Skirball 8R, New York, NY 10016 USA; 4 Division of Hematology/Oncology, Department of Pediatrics, NYU Grossman School of Medicine, NYU Langone Health, 160 E 32nd St, New York, NY 10016 USA; 5 Hansjörg Wyss Department of Plastic Surgery, NYU Grossman School of Medicine, NYU Langone Health, 305 E 33rd St, New York, NY 10016 USA; 6 Department of Pathology, NYU Grossman School of Medicine, NYU Langone Health, 550 First Ave, MSB 5th Floor, New York, NY 10016 USA; 7 Department of Ophthalmology, NYU Grossman School of Medicine, NYU Langone Health, 222 E 41st St, 3rd and 4th Floors, New York, NY 10017 USA

**Keywords:** IgG4-related disease, Rosai-Dorfman disease, Orbit, Inflammatory lesion

## Abstract

Inflammatory orbital lesions include a broad list of diagnoses, many of them with overlapping clinical and radiographic features. They often present a diagnostic conundrum, even to the most experienced orbital specialist, thus placing considerable weight on surgical biopsy and histopathological analysis. However, histopathological diagnosis is also inherently challenging due to the rarity of these lesions and the overlaps in histologic appearance among distinct disease entities. We herein present the case of an adolescent male with a subacutely progressive orbital mass that generated a significant diagnostic dilemma. Early orbital biopsy was consistent with a benign fibro-inflammatory lesion, but corticosteroid therapy was ineffective in halting disease progression. After an initial substantial surgical debulking, histopathological analysis revealed several key features consistent with IgG4-related disease (IgG4-RD), a systemic fibro-inflammatory process typically accompanied by multifocal tumor-like lesions. Surprisingly, within months, there was clear evidence of clinical and radiographic disease progression despite second-line rituximab treatment, prompting a second surgical debulking. This final specimen displayed distinctive features of Rosai-Dorfman disease (RDD), a systemic inflammatory disease characterized by uncontrolled histiocytic proliferation. Interestingly, certain features of this re-excision specimen were still reminiscent of IgG4-RD, which not only reflects the difficulty in differentiating RDD from IgG4-RD in select cases, but also illustrates that these diagnoses may exist along a spectrum that likely reflects a common underlying pathogenetic mechanism. This case emphasizes the importance of surgical biopsy or resection and histopathological analysis in diagnosing—and, ultimately, treating—rare, systemic inflammatory diseases involving the orbit, and, furthermore, highlights the shared histopathological features between RDD and IgG4-RD.

## Introduction

Orbital lesions can be grouped under six general categories: inflammatory, infectious, vascular, neoplastic, metastatic (or secondarily invading), and developmental [1]. Whereas some entities—particularly vascular orbital lesions such as capillary hemangioma—may feature unique characteristics on imaging, most others share clinical and radiographic features, which complicates the establishment of a definitive diagnosis [1–3]. Distinguishing between inflammatory masses and hematological lesions (in particular, lymphoma) based solely on clinical and radiographic findings is especially difficult [3]. Additionally, the inflammatory syndromes that can present with orbital masses are themselves a diverse category with many overlapping features, including clinical history, physical examination, and imaging findings [3, 4]. In children and adolescents especially, inflammatory orbital masses are rare and present a significant diagnostic challenge [4]. These considerations point towards the importance of both obtaining substantive biopsy or surgical specimens and detailed histopathological analysis in making a definitive diagnosis of such lesions [3].

We present the case of an adolescent male with an inflammatory orbital mass that was especially challenging to diagnose. He presented with a subacute, progressive, inflammatory left orbital mass that was given a histopathological diagnosis of IgG4-related disease (IgG4-RD) following initial surgical debulking. However, the lesion failed to respond to both first- and second-line treatment for IgG4-RD and required further debulking due to disease progression. Biopsy specimens obtained following a second surgical debulking revealed histology that ultimately prompted a final diagnosis of Rosai-Dorfman disease (RDD).

## Case presentation

**Fig. 1:**
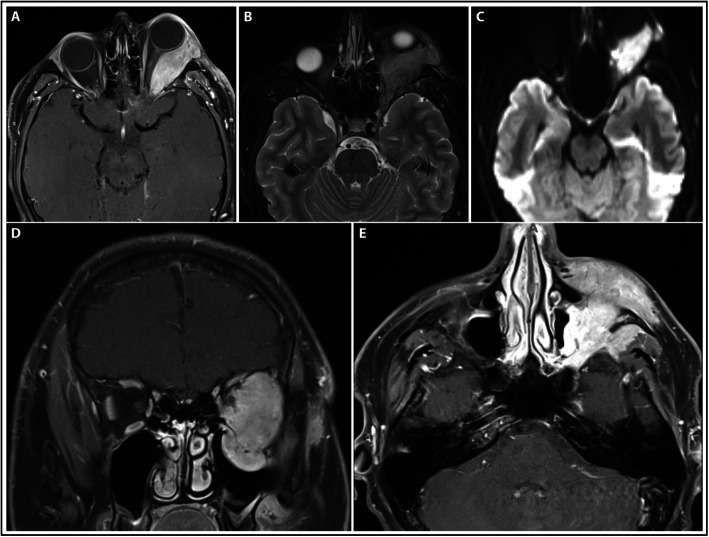
MRI brain and orbits after multiple biopsies and worsening of ophthalmic symptoms. **a** T1-weighted post-contrast axial sequence at the level of the optic nerve demonstrating avid enhancement of the left orbital lesion with infiltration of the left lateral rectus muscle and obvious proptosis without associated optic nerve enhancement. **b** T2-weighted axial sequence showing the lobulated left orbital mass to be hypointense and without significant surrounding edema. **c** Diffusion-weighted imaging showing significant lesional diffusion restriction. **d** Coronal and (**e**) additional axial T1-weighted post-contrast images showing extension of the homogeneously-enhancing mass into the left maxillary sinus, pterygopalatine fossa, infratemporal fossa, and surrounding soft tissues

The patient was an otherwise healthy 17-year-old male who presented with left eyelid swelling, retrobulbar pressure sensation, and occasional diplopia worsening over the preceding few weeks. MRI revealed an infiltrative, enhancing, multi-compartmental lesion in the left inferolateral extraconal orbit associated with left-sided proptosis, optic nerve displacement, suspected osseous erosion of the orbital floor, and nodular lesions along the left frontal scalp and buccal region. Initial transconjunctival orbital biopsy was suggestive of a non-specific, benign, fibro-inflammatory lesion, but oral prednisone therapy resulted only in minimal symptom improvement without significant radiographic changes. An additional minimally invasive periorbital biopsy was more consistent with an atypical lymphoid infiltrate with prominent B cell and polytypic plasma cell populations—considered potentially concerning for marginal zone lymphoma. Of note, PET-CT imaging was negative for evidence of lymphoma elsewhere. During the next several months, the patient’s ophthalmic symptoms progressed, and he developed new neurological symptoms (facial numbness and tingling). Interval enlargement of the orbital lesion was noted on repeat MRI (Fig. 1a-e).

Initial surgical debulking of the left intraorbital retrobulbar mass was performed by a multidisciplinary surgical team including skull base and pediatric neurosurgeons and plastic surgery faculty; via a left orbitozygomatic craniotomy, the bulk of the lesion bordering the extraocular muscles and the portion extending into the infratemporal fossa was resected and sent for histopathological analysis. A fibroinflammatory process characterized by a dense lymphohistiocytic infiltrate (Fig. 3a) containing abundant plasma cells (Fig. 3c) was observed. Immunohistochemical staining for IgG and IgG4 highlighted a large subpopulation of IgG4-positive plasma cells (greater than 50 per high-power field) (Fig. 3f), and the overall IgG4/IgG expression ratio was greater than 0.4 (Fig. 3e-f). Vague areas of storiform fibrosis (Fig. 3b) and focal obliterative phlebitis (not shown) were also identified. Immunostaining for ALK-1, which is useful when trying to establish a diagnosis of inflammatory myofibroblastic tumor, was negative (Fig. 3d). Taken together, these features favored the diagnosis of IgG4-RD.

**Fig. 2:**
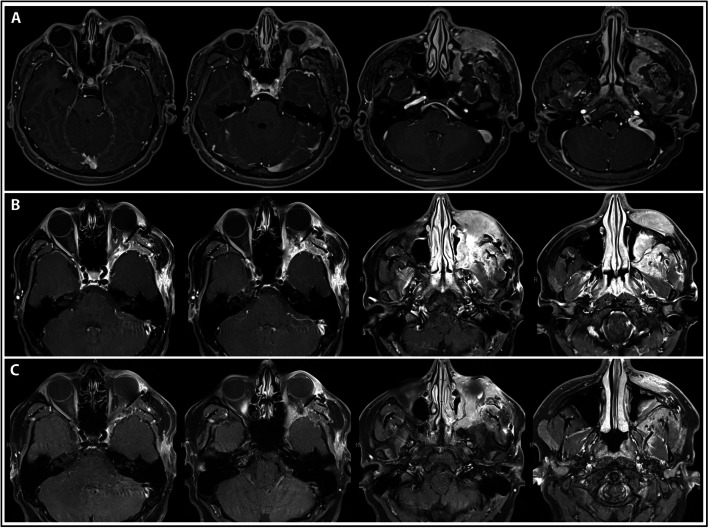
Postoperative and follow-up MRI brain and orbits after initial and second surgical debulkings. **a** Postoperative T1-weighted post-contrast MRI brain and orbits obtained after initial surgical debulking demonstrating significant resection at the level of the optic nerve with residual enhancing mass in the infraorbital region, left cheek, and maxillary sinus. There is residual circumferential enhancement around the optic nerve at the orbital apex. A small postoperative fluid collection along the lateral and inferior left orbit is also observed. **b** Follow-up T1-weighted post-contrast MRI obtained approximately 2 months after initial surgical debulking showing increased lobular enhancing soft tissue mass protruding through the left orbital floor into the left maxillary sinus. Increased enhancement in the left retromaxillary fat and along the left maxillary alveolar ridge are consistent with progressive disease. **c** Follow-up MRI obtained approximately 1 month after second surgical debulking demonstrating interval resection of the premaxillary and infraorbital soft tissue enhancement without evidence of disease progression

Postoperative imaging revealed radical resection at the level of the optic nerve with residual enhancing mass in the infraorbital region, the left cheek, and the maxillary sinus (Fig. 2a). The patient continued to experience left eyelid swelling, diplopia, and cheek numbness. He received two doses of second-line rituximab (since he previously failed first-line corticosteroids), but despite this, an MRI obtained approximately 2 months after initial surgical debulking revealed extracranial disease progression in the maxillary sinus and fat planes of the left cheek (Fig. 2b).

**Fig. 3:**
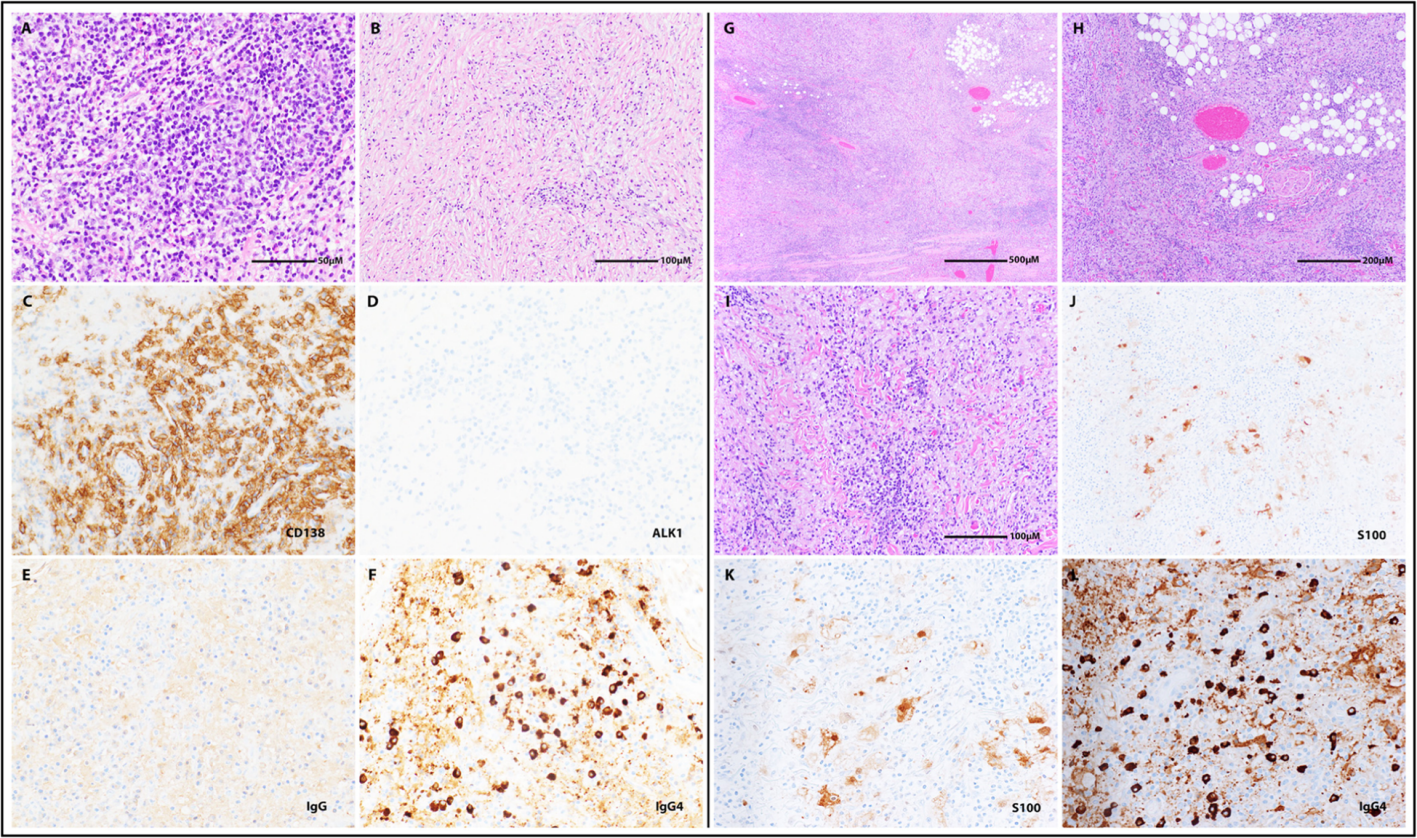
Histopathological findings after initial and second surgical debulkings. **a**-**f** Histopathological findings after initial surgical debulking. Scale bar found in (**a**) is applicable to all panels unless otherwise indicated. **a** H&E, high-power, lymphohistiocytic inflammatory infiltrate with abundant plasma cells. **b** H&E, low-power, significant storiform fibrosis. **c** Inflammatory infiltrate is composed predominantly of plasma cells highlighted by CD138. **d** No ALK-1 immunopositivity was observed. **e** IgG and (**f**) IgG4 immunostains revealed > 50 IgG4-positive plasma cells per high-power field and an IgG4/IgG ratio of > 0.5. **g**-**l** Histopathological findings after second surgical debulking: (**g**) H&E, low-power, diffuse inflammatory infiltrate composed of small lymphocytes, histiocytes, and plasma cells with entrapped fat and adjacent skeletal muscle. **h** H&E, medium-power, highlighting vasculature without evidence of obliterative phlebitis (also not seen with elastic stain, not shown). **i** H&E, high-power, inflammatory infiltrate with collagen bands; absence of sclerosis. Histiocytes demonstrate S100 immunopositivity at (**j**) medium-power (scale bar in (**i**) applies) and (**k**) high-power (scale bar in (**a**) applies), and emperipolesis is noted within the larger histiocytes. **l** The plasma cell component is rich in IgG4, up to 50–60 per high-power field; the IgG/IgG4 ratio is approximately 0.3 (IgG immunostain not shown)

Therefore, nearly three months after the initial debulking, a second debulking surgery of the progressive extracranial disease was attempted by plastic surgery; dissection and en bloc resection of the mass from the caudal margin of the tarsal plate down into the cheek, around the deep muscles of the face, and through to the deep extension to the infraorbital fissure was performed, and specimens were again sent to pathology. Surprisingly, histopathological findings of this second surgical specimen revealed new, informative diagnostic features. While a diffuse inflammatory infiltrate characterized by small lymphocytes, histiocytes, and IgG4-positive plasma cells (up to 50–60 per high-power field) was still present (Fig. 3g-i, l) and was associated with a somewhat elevated plasma cell IgG4/IgG expression ratio (0.3), there was a lack of apparent storiform architecture in the fibrous stroma, and obliterative phlebitis was absent (Fig. 3h-i). Furthermore, large S-100-positive histiocytes with foamy cytoplasm and hypochromatic nuclei with prominent nucleoli were newly observed, and there was evidence of emperipolesis (Fig. 3j-k). Although the inflammatory component seen here was reminiscent of IgG4-RD given its similarity to the previous specimen, new findings more strongly supported a different final diagnosis: RDD.

**Fig. 4:**
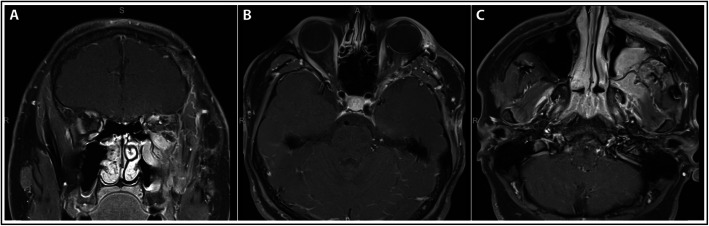
Follow-up MRI brain and orbits after second surgical debulking. Follow-up T1-weighted post-contrast MRI brain and orbits obtained approximately 4 months after second surgical debulking showing stable residual disease in the superior left maxillary sinus and along the left orbital floor involving the inferior rectus muscle in the (**a**) coronal plane, (**b**) axial plane at the level of the optic nerve, and (**c**) axial plane at the level of the maxillary sinus. Notably, the globes are normal in contour and there is resolution of prior proptosis

Following extracranial debulking, there was persistent multi-directional diplopia; however, MRIs obtained at approximately 1 month (Fig. 2c) and 4 month (Fig. 4) follow-up showed interval decrease in lesion size consistent with surgical resection, and stabilization of disease thereafter, including resolution of proptosis. Post-surgical cicatricial ectropion of the left lower eyelid along with myogenic ptosis of the left upper eyelid were subsequently repaired by oculoplastic surgery. No further treatment was required.

## Discussion and conclusions

This case of orbital, extranodal RDD with features reminiscent of IgG4-RD presented a diagnostic dilemma that highlights the subtleties in diagnosing either disease in an atypical location and supports the potential existence of an overlap syndrome.

The differential diagnosis before surgical debulking was broad. One possibility was inflammatory myofibroblastic tumor (IMT), which tends to occur in children and can present as a well-defined soft tissue mass (as initially seen in our patient) [5, 6]. Although uncommon, orbital IMT has been reported in both children and adults; its clinical features (unilaterality, proptosis, diplopia, periocular swelling, and others) are often similar to our patient’s [5, 7, 8]. Ocular adnexal lymphoma was also on the differential, as patients can present with exophthalmia, orbital mass, reduced visual acuity, and diplopia [9]. Orbital meningioma, Graves’ orbitopathy, and idiopathic orbital inflammation, although less likely, were also considered [10, 11].

Histopathological analysis following the initial surgical debulking of the lesion’s orbital portion revealed several key features, described above and shown in Fig. 3a-f, that met consensus criteria (delineated by Deshpande et al. in 2012) for tissue diagnosis of IgG4-RD [12]. Also supporting this diagnosis was the presence of myofibroblasts and dense regions of IgG4-immunopositive plasma cells. IgG4-RD typically presents as a systemic fibro-inflammatory process accompanied by multifocal tumor-like lesions [12]. Although type 1 autoimmune pancreatitis is the archetypal manifestation, the head and neck region is the second most commonly affected area [13, 14]. The first systematic review of head and neck IgG4-RD found that the orbit was the most frequently affected site [15, 16]. Interestingly, an additional systematic review of pediatric IgG4-RD found that primary orbital disease was the most common manifestation in children [17, 18]. Orbital IgG4-RD in adults usually involves the lacrimal gland; however, in children, the surrounding orbital soft tissues, musculature, and trigeminal nerve branches are more typically involved (as seen in our patient) [15, 18]. First-line IgG4-RD treatment is typically 2–4 weeks of steroids (usually prednisone) followed by a gradual taper [19]. In the rare case that steroids fail to control disease, rituximab is considered second-line treatment [20]. A systematic review of treatment outcomes in 95 patients with orbital IgG4-RD suggested that rituximab is the most efficacious non-steroidal immunosuppressive agent, with a success rate greater than 90% [21]. The failure of our patient’s lesion to respond to both steroids and rituximab, while possible in IgG4-RD, is extremely unusual and questioned the validity of this initial diagnosis.

Histopathological analysis following the second debulking surgery shifted our diagnosis towards RDD. RDD is an inflammatory syndrome characterized by uncontrolled histiocytic proliferation [22]. It is rare, with an incidence in the United States of approximately 100 cases annually [23]. While extranodal RDD is identified in approximately 43% of cases, ophthalmic presentations have been reported in only 11% [22]. Facial cutaneous RDD is even rarer, and interestingly, most facial cutaneous RDD manifestations (including those seen in the periorbital soft tissues) present as isolated lesions [24]. Age at presentation is skewed towards younger individuals (such as our patient), with a mean age at diagnosis of approximately 21 years [22]. Males and/or those of African descent are disproportionately affected [23]. RDD treatment varies by affected site, ranging from observation alone in simple lymphadenopathy or asymptomatic cutaneous RDD to steroids for more symptomatic, progressive, or multifocal disease—although steroid efficacy is not well-established [23].

The biopsy specimens obtained from the second surgical debulking were most notable for having numerous “RDD cells”: large, S-100-positive histiocytes with “watery-clear” or “foamy” cytoplasm and a hypochromatic nucleus with a conspicuous nucleolus (Fig. 3k) [25]. Emperipolesis, another canonical feature of RDD, was also observed (Fig. 3k) [23]. Emperipolesis is less conspicuous in cutaneous manifestations of RDD; in equivocal inflammatory facial lesions, larger excisional biopsy specimens seem necessary to better ensure comprehensive analysis of such features [24]. Some features of IgG4-RD, such as an elevated plasma cell IgG4/IgG expression ratio, were still present; however, in this specimen, the ratio did not strictly meet IgG4-RD diagnostic criteria, and both storiform fibrosis and obliterative phlebitis were absent as well. Local recurrence of RDD following surgical resection—particularly in orbital disease, as in this case—is not uncommon [26–28]. Ultimately, local disease progression despite steroid and rituximab treatment, the need for extensive surgical debulking to achieve disease stabilization, new histopathological findings, and a clinical picture consistent with RDD converged onto a final unifying diagnosis of extranodal RDD, notably with focal atypical features reminiscent of IgG4-RD.

Interestingly, a report published by Bertero et al. described a similar case of an orbito-temporal mass ultimately diagnosed as “Rosai-Dorfman meningeal disease with IgG4-related disease histological features”; just as in our case, initial histopathological analysis of was suggestive of IgG4-RD, while further samples exhibited features pathognomonic for RDD [29]. The histologic overlap between IgG4-RD and RDD in both of these cases may indicate a common pathogenesis underlying at least a subset of these two inflammatory processes. Kuo et al. first proposed a potential relationship between IgG4-RD and RDD based on shared histological and molecular features across 12 cutaneous RDD cases [30]. In addition to the presence of pathognomonic “RDD cells,” all 12 cases demonstrated abundant IgG4-positive plasma cells and various degrees of stromal fibrosis. Furthermore, three cases in this series met strict consensus criteria for plasma cell expression of IgG4 for IgG4-RD established by Deshpande et al. [12], with an IgG4/IgG ratio greater than 0.4 [30]. Fayne et al. provide another cases series also suggesting that, especially in primary cutaneous manifestations of RDD, there exists a potential subgroup with an abundance of plasma cells and inconsistent RDD features throughout the lesion (particularly emperipolesis) [31]. In a larger series of 26 RDD samples, which included both nodal and extranodal cases, Zhang et al. found that 30.8% of cases displayed an IgG4/IgG ratio that met consensus criteria for IgG4-RD [32]. Additionally, they demonstrated that IgG4 plasma cell burden and regulatory T-cell (T-reg) infiltration were positively correlated with the degree of lesional sclerosis, particularly in recurrent or persistent disease [32]. T-regs produce interleukin-10 (IL-10) and transforming growth factor-β (TGF-β); the former induces IgG4 class switching, and the latter induces collagen production and fibrosis—both critical phenomena in IgG4-RD pathogenesis [33]. The variable presence of T-regs and subsequent IgG4 expression in RDD, particularly in extranodal progressive disease (as seen in our patient), suggests either that RDD and IgG4-RD may coexist across a single pathological spectrum, or that RDD may demonstrate characteristics of IgG4-RD during specific phases of disease progression as a consequence of T-reg influence on associated inflammation.

Another key observation from Zhang et al. was that adult RDD cases harbored more sclerosis than pediatric cases [32]. Two additional series replicated this association of older age at presentation with increased sclerosis and IgG4-RD features [34, 35]. In a series of 32 nodal and extranodal specimens reviewed by Liu et al., three demonstrated an IgG4/IgG ratio greater than 0.4—all from older adolescents or adult patients [34]. Menon et al., who reviewed the largest RDD series to date of 70 samples for features of IgG4-RD, also found that 12 cases met consensus criteria for IgG4 expression; the median age at presentation of these 12 cases and the median age at presentation of all IgG4-positive cases in the series (55 and 54 years, respectively) were both significantly higher than that for IgG4-negative cases, at 27 years [35]. Menon et al. also identified a significant male predominance among the highest IgG4-expressing cases [35]. Presentation of cutaneous RDD with features of IgG4-RD in an older adolescent male fits with the limited clinical and histopathological data on this potential overlap syndrome. The seemingly temporal nature of the presentation of RDD with IgG4-RD features is furthermore suggestive of a spectrum disorder, with pathogenesis potentially varying according to age of onset in addition to the likely varied contribution of T-regs. Identification of RDD cases that lean towards IgG4-RD may also have critical implications for treatment. Most RDD cases undergo spontaneous remission [36], but a handful of reports on refractory cases—in particular, those with IgG4 immunopositivity—have noted unusual steroid-responsiveness [32, 37]. Since IgG4-RD and related syndromes are known to respond dramatically to steroid treatment [38], further investigation into IgG4’s role as a potential biomarker for steroid-responsiveness in RDD is warranted, although this phenomenon was not immediately apparent after a short course of steroids in this patient’s case.

The diagnostic dilemma highlighted by this case is critically hypothesis-generating with respect to the nature of adult extranodal RDD and its potential relationship with IgG4-RD. Informative histopathological evidence was not obtainable without substantial surgical tissue sampling. Furthermore, the patient’s lesion appeared to display not only regional differences in plasma cell and histiocyte expression, but also a clinical response to treatment not clearly consistent with any previously reported RDD-related or IgG4-RD–related entity. Previous series have identified examples of RDD with features of IgG4-RD, especially in young adults with progressive, refractory disease. Further investigation into whether RDD and IgG4-RD may exist across a shared pathological spectrum due to similar molecular pathogenesis, likely dependent on T-regs, is necessary to improve our understanding and optimize our management of these rare and challenging systemic diseases.

## Data Availability

There are no associated datasets for this manuscript. Related queries can be directed to the corresponding author.

## Abbreviations

IgG4-RD: IgG4-related disease
RDD: Rosai-Dorfman disease
IMT: Inflammatory myofibroblastic tumor
T-reg: Regulatory T-cell
IL-10: Interleukin-10
TGF-β: Transforming growth factor-β
